# Genotype-phenotype insights of pediatric dilated cardiomyopathy

**DOI:** 10.3389/fped.2025.1505830

**Published:** 2025-01-31

**Authors:** Ying Dai, Yan Wang, Youfei Fan, Bo Han

**Affiliations:** Department of Pediatrics, Shandong Province Hospital Affiliated to Shandong First Medical University, Jinan, Shandong, China

**Keywords:** children, dilated cardiomyopathy, genetics, inherited cardiomyopathy, genotype-phenotype correlation

## Abstract

Dilated cardiomyopathy (DCM) in children is a severe myocardial disease characterized by enlargement of the left ventricle or both ventricles with impaired contractile function. DCM can cause adverse consequences such as heart failure, sudden death, thromboembolism, and arrhythmias. This article reviews the latest advances in genotype and phenotype research in pediatric DCM. With the development of gene sequencing technologies, considerable progress has been made in genetic research on DCM. Research has shown that DCM exhibits notable genetic heterogeneity, with over 100 DCM-related genes identified to date, primarily involving functions such as calcium handling, the cytoskeleton, and ion channels. As human genomic variations are linked to phenotypes, DCM phenotypes are influenced by numerous genetic variations across the entire genome. Children with DCM display high genetic heterogeneity and are characterized by early onset, rapid disease progression, and poor prognosis. The genetic architecture of pediatric DCM markedly differs from that of adult DCM, necessitating analyses through clinical phenotyping, familial cosegregation studies, and functional validation. Clarifying the genotype-phenotype relationship can improve diagnostic accuracy, enhance prognosis, and guide follow-up treatment for genotype-positive and phenotype-negative patients identified through genetic testing, providing new insights for precision medicine. Future research should further explore novel pathogenic genes and mutations and strengthen genotype-phenotype correlation analyses to facilitate precise diagnosis and treatment of DCM in children.

## Introduction

Dilated cardiomyopathy (DCM) in children is a myocardial disease characterized by enlargement of the left ventricle or both ventricles, accompanied by systolic dysfunction (1). DCM is one of the most common causes of heart failure in children, accounting for approximately 30%–50% of cases (2). Pediatric DCM progresses rapidly and has a poor prognosis, with a 5-year survival rate of only 50%–60% and with most deaths resulting from progressive heart failure and its complications (3). The etiology of pediatric DCM is diverse and includes genetic, infectious, metabolic, toxic, and idiopathic causes. Approximately 30%–50% of pediatric patients with DCM have pathogenic gene mutations, primarily involving genes encoding sarcomere proteins, ion channels, Z-disc proteins, and nuclear envelope proteins (4). Currently, according to the Human Gene Mutation Database and Online Mendelian Inheritance in Man database,over 100 genes have been identified to be associated with monogenic hereditary DCM among the pathogenic genes in hereditary/familial DCM. These include *TTN, LMNA, DSP, PLN, FLNC, RBM20, SCN5A, MYH7, MYBPC3*, and others (5, 6). However, after in-depth data analysis, the associations of some of these genes with DCM were not supported. In recent years, with advancements in research on additional genes and genotype-phenotype correlations, phenotypic overlap and dynamic changes in DCM phenotypes have been discovered. Genetic heterogeneity makes it challenging to accurately classify DCM and guide clinical decision-making, thus posing challenges for traditional DCM diagnostic methods (7, 8). There are both similarities and significant differences between DCM and hypertrophic CM (HCM). There is notable phenotypic overlap between arrhythmogenic CM (ACM) and DCM, with specific genes such as *LMNA, SCN5A, FLNC, RBM20, PLN, DSP*, and *DES* potentially causing ACM (9). Clarifying genotype-phenotype relationships can enable rapid and precise interpretation of candidate variants in pediatric DCM and greater understanding of its specific variant spectrum, which is crucial for guiding clinical treatment strategies and developing new therapeutic approaches (10). Although understanding of the genotypes and phenotypes of pediatric DCM continues to deepen, several issues remain unresolved. This article reviews the latest advances in genotype and phenotype research in pediatric DCM and discusses future research directions with the aim of providing new insights for precision medicine in pediatric DCM.

**Figure 1:**
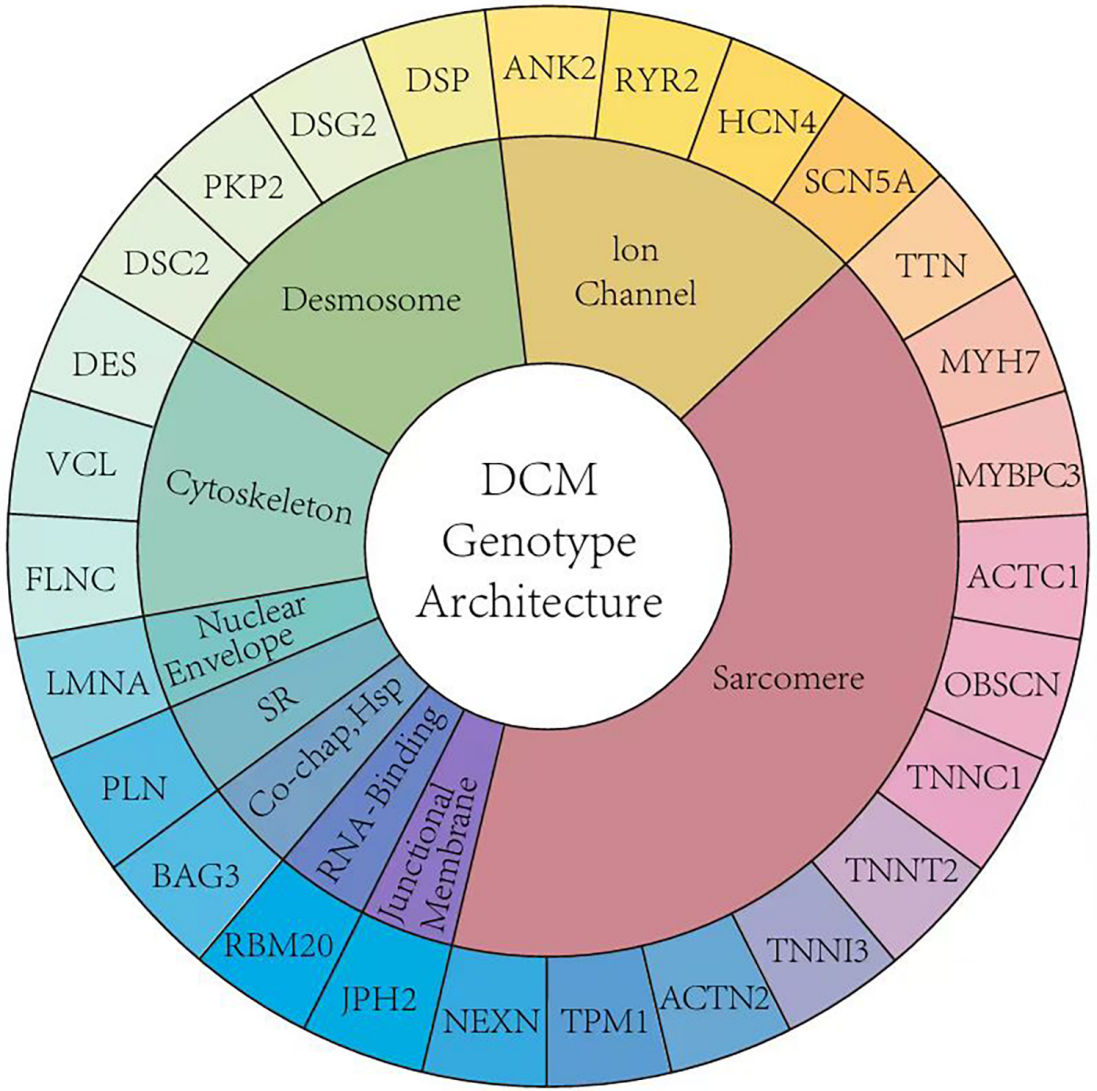
The genetic architecture of pediatric DCM.

**Figure 2:**
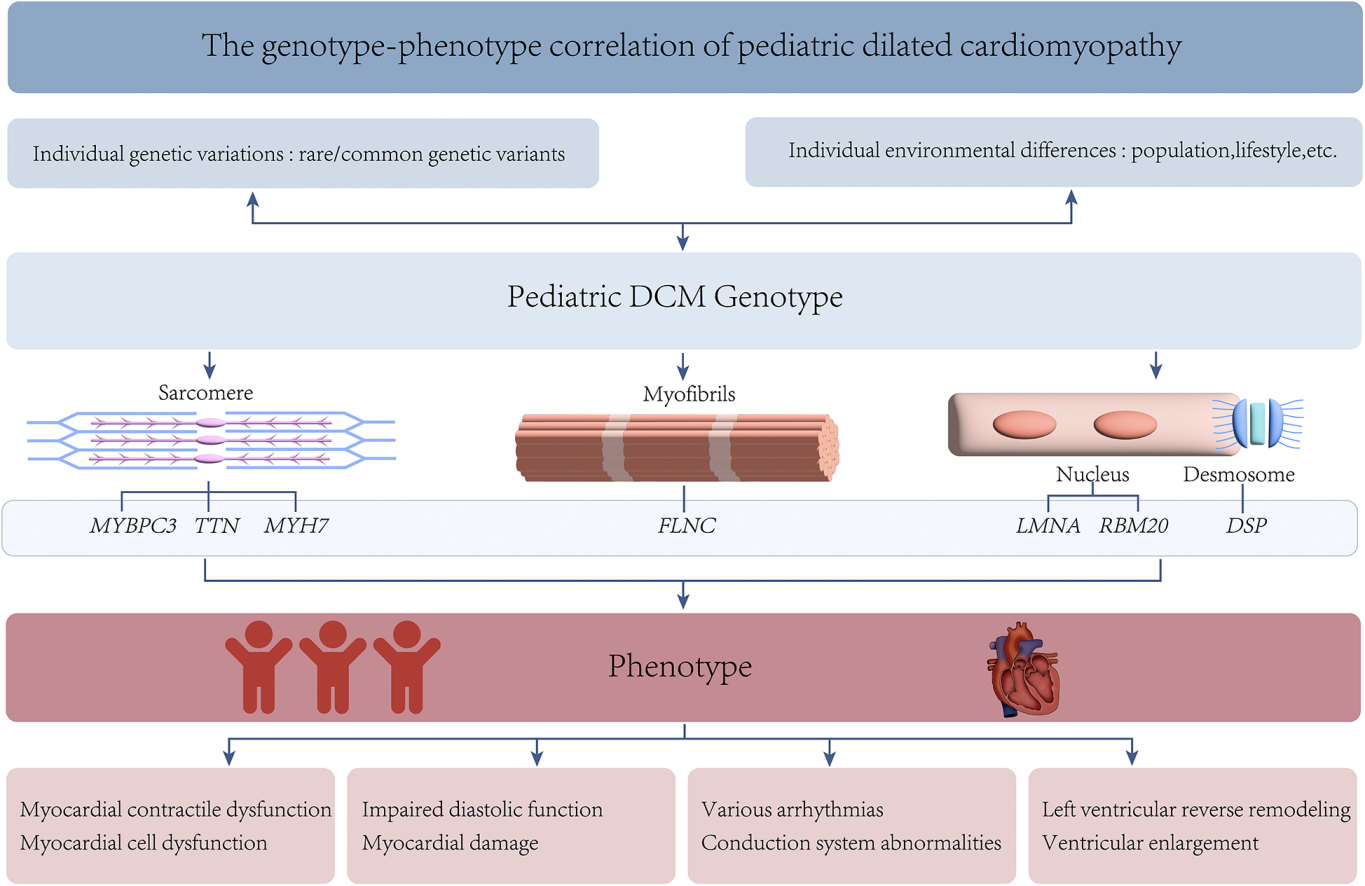
The genotype and phenotype of pediatric DCM.

**Table 1:** Genotype and related phenotype in pediatric dilated cardiomyopathy.

Functional Group	Gene symbol	Gene Name	Mode of inheritance	Classification	Phenotype	Ref.
Sarcomere	*TTN*	Titin	AD	Definitive	Cardiomyocyte dysfunction, Left ventricular reverse remodeling, Neuromuscular disorders, Myopathy	(18, 73, 81, 87)
	*MYH7*	Myosin Heavy Chain 7	AD	Definitive	Myocardial contractile dysfunction, Left ventricular reverse remodeling, Heart failure	(12, 66, 87)
	*MYBPC3*	Myosin Binding Protein C 3	AD	Limited	Systolic and diastolic dysfunction	(26)
Nuclear Envelope	*LMNA*	Lamin A/C	AD	Definitive	Arrhythmias and conduction system disorders, Sudden cardiac death, Neuromuscular disorders, Myopathy	(32, 33, 80)
Cytoskeleton	*FLNC*	Filamin C	AD	Definitive	Muscle cell dysfunction, Ventricular arrhythmias, Myocardial fibrosis, Sudden cardiac death, Left ventricular regional wall motion abnormalities	(36, 37, 79)
	*DES*	Desmin	AD	Definitive	Muscle cell dysfunction	(9, 13)
Ion channels	*SCN5A*	Sodium Voltage-Gated Channel Alpha Subunit 5	AD	Definitive	Ventricular enlargement, Decreased myocardial contractility, Arrhythmia	(42, 43)
Desmosomes	*DSP*	Desmoplakin	AD	Definitive	Skin and hair abnormalities, Malignant arrhythmia	(47, 79)
Sarcoplasmic reticulum	*PLN*	Phospholamban	AD	Moderate	Arrhythmia	(9)
Z-disk	*BAG3*	BAG Cochaperone 3	AD	Definitive	Skeletal muscle disorders	(13, 14)
Transcriptional regulation	*RBM20*	RNA Binding Motif Protein 20	AD	Definitive	Early-onset heart failure, Ventricular arrhythmia	(51–53)

## Genetic architecture of pediatric DCM

When comparing the genetic composition of DCM in children and adults, although the guidelines recommend genetic testing, there are significant differences in actual clinical practice (Figure 1). In DCM, the presence of variants of uncertain significance (VUS) alone [odds ratio [OR] 4.0, 95% confidence interval [CI] 1.9–8.3] and in combination with pathogenic variants (OR 5.2, 95% CI: 1.7–15.9) is associated with major adverse cardiac events (11). Studies have shown that children with cardiomyopathy have a higher incidence of neuromuscular diseases, congenital metabolic defects, mitochondrial diseases, and malformation syndromes (12). However, due to the lack of large-scale clinical studies, understanding of the genetic composition of pediatric DCM is limited, and the existence of child-specific DCM pathogenic genes cannot be ruled out. Pediatric DCM exhibits high genetic heterogeneity with a higher number of rare variants, most of which are VUS. This necessitates analyses using clinical phenotyping, familial cosegregation studies, and functional validation (13). A Finnish single-center study conducted a 20-year follow-up of 66 children with DCM and found that 39% of the cases had at least one disease-related gene mutation. These diseases included metabolic disorders, sarcomere-related diseases, and various other syndromes. The predominant mutations were missense variants (91.5%) and loss-of-function mutations (5.4%). This Finnish single-center study reported that *NRAP* is a new cause of severe DCM and other atypical clinical phenotypes in children. *PPA2* deficiency involving sudden death may lead to DCM, and junctophilin-2 variants may cause recessive DCM with childhood onset (12).

Mutations in the sarcomere-related genes (*TTN, MYH7*, and *MYBPC3*) are common in DCM. Studies have found that approximately 10%–13% of patients aged 2–18 years have *TTN* variants. In DCM cohorts, mutations in genes such as *BAG3, CRYAB*, and *DES* can be observed, with some children showing evidence of skeletal muscle involvement (13, 14). These data indicate genotype-phenotype correlations, emphasizing the importance of genotype-directed therapy in pediatric DCM. However, Canadian literature suggests that the incidence of *TTN*-truncating variants (*TTN*tv) is higher in adult DCM patients than in pediatric DCM patients. Compared to adults, pediatric DCM patients have a lower variant burden of channelopathy genes (15).

In a North American multi-center study, genomic testing was conducted on 279 pediatric and adolescent patients with DCM (<18 years old) from 14 medical institutions in the United States and Canada. In this pediatric DCM cohort, no pathogenic/likely pathogenic gene had a frequency exceeding 4%. The frequencies of pathogenic/likely pathogenic variants of *MYH7, MYBPC3, TNNT2*, and *RBM20* were similar to those in adult DCM. However, the frequencies of variants in *TTN* and *LMNA* were lower than those in adult patients with DCM (13).

A retrospective analysis was conducted on 299 pediatric patients with DCM (aged < 18 years) who received treatment at the Children's Hospital of Chicago in the United States between 2007 and 2016. The study found that the genetic architecture differed from that of adults; 37% of pediatric patients with DCM had pathogenic/likely pathogenic gene mutations in sarcomere-related genes. Mutations in *LMNA*, *RBM20*, and *PLN*, which are common in adult DCM, were found to have a lower frequency in pediatric DCM. Additionally, the study emphasized the age-dependent risk associated with *TTN*tv), which were associated with a later age of onset (average age at initial diagnosis: 9.7 years). *TTN*tv carriers had a poor prognosis, 60% of patients carrying *TTN*tv ultimately die or undergo heart transplantation (14).

## Genotype insights in relation to pediatric DCM

Pediatric DCM is characterized by enlargement of the left or both ventricles and reduced contractile function (16). *TTN* is located on chromosome 2q31 and contains 364 exons. Extensive mRNA splicing can produce various titin isoforms, with N2B and N2BA being the main cardiac-related isoforms (17). *TTN* encodes titin, which is the largest protein in the muscles and is crucial for the structure and function of cardiomyocytes (18). *TTN* mutations have the highest detection rate in pediatric patients with DCM and are an important genetic factor in pediatric DCM (19). *TTN*-related DCM usually follows an autosomal-dominant inheritance pattern; however, complex inheritance patterns also exist (20), with *TTN*tv being the most common (21). *TTN*tv mutations occur predominantly in the A-band region, with smaller proportions occurring in the I-band, Z-disc, and M-line regions. Children with *TTN*tv mutations in the A-band or M-line regions have a poor prognosis (17). DCM caused by *TTN* mutations exhibits significant phenotypic variability. Even within the same family, there are considerable differences in disease severity and age of onset (22). In adult patients with DCM, *TTN*tv are not uniformly distributed but rather cluster in the A-band region (*p* = 3.4 × 10^−4^*p* = 3.4 × 10^−4^) (*p* = 3.5 × 10^−3^−3*p* = 3.5 × 10^−3^). However, this clustering is not observed in pediatric patients (15).

*MYH7* encodes the myosin protein, which consists of 1,935 amino acids. Myosin is primarily expressed in myocardium and type 1 skeletal muscle fibers and is a important component of the human ventricular system. It plays a crucial role in supplying energy to cardiomyocytes and in maintaining intracellular and extracellular Ca2+ concentrations (23). *MYH7* mutations can impair myocardial contractile function, resulting in DCM (24). In pediatric patients, pathogenic variants in the *MYH7* gene are not evenly distributed but mainly cluster in the myosin head and neck regions (Kolmogorov–Smirnov goodness of fit test: *p* = 8.4 × 10^−4^, *p* = 8.4 × 10^−4^). In adults, variants are primarily concentrated in the myosin head and neck regions, but also appear in the tail region, similar to the reference population (15).

*MYBPC3* encodes myosin-binding protein C and is the second most common pathogenic gene in DCM after *MYH7*. It plays a crucial role in maintaining myocardial sarcomere structure and regulating myocardial contraction (25). Mutations in *MYBPC3* can lead to the abnormal binding of protein C, resulting in myocardial contraction and relaxation dysfunction (26). *TNNT2* and *TNNI3* encode troponins T and I, respectively. These genes encode key proteins involved in myocardial contractions. Mutations in these genes typically lead to impaired myocardial contractile function, thereby causing DCM (27, 28). *TPM1* encodes the cardiac-specific α-chain of tropomyosin. Mutations in this gene affect the assembly and function of myofilaments, leading to decreased myocardial contractility (29). Variants in *MYBPC3* cluster mainly in the C5, C7, and C10 regions, with no significant differences between children and adults (15).

*LMNA* is located on chromosome 1q21.1–21.3, and contains 12 exons. It encodes type A nuclear lamins, primarily lamins A/C. Vertebrate nuclear lamins include A and B forms. Human lamin A/C produces three type A isoforms through alternative splicing (30, 31). Mutations in *LMNA* can lead to various cardiac diseases, including DCM, arrhythmias, and conduction system diseases (32). *LMNA*-related DCM typically has an earlier age of onset, faster disease progression, and poorer prognosis. DCM caused by *LMNA* mutations is often accompanied by arrhythmias and conduction system abnormalities, which increase the risk of sudden death in patients (33). Currently, the treatment for *LMNA*-related DCM includes standard heart failure therapy and implantable cardioverter-defibrillator (ICD) implantation to prevent sudden death. *LMNA* is the only gene in the current guidelines with a Class I recommendation for ICD implantation (34).

*FLNC* encodes the actin-binding protein filamin C, which plays important structural and signaling roles in muscle cells (35). Mutations in *FLNC* can lead to abnormal myofilament structures and muscle cell dysfunction, resulting in cardiomyopathy (36). Studies have shown that *FLNC* mutations are significantly associated with pediatric DCM. Compared to the average clinical course of DCM, *FLNC*-related DCM is more malignant and characterized by a high risk of ventricular arrhythmias, myocardial fibrosis, and sudden cardiac death. The average age of onset is 39.7 ± 14.5 years, and *FLNC*-related DCM is more commonly found in adults (37). However, a pediatric study discovered biallelic *FLNC* variants in a family with previously unreported pediatric DCM. Biallelic *FLNC* variants can lead to congenital DCM (38). Understanding the role of *FLNC* may provide new insights for the development of targeted therapeutic strategies (39).

*SCN5A* encodes the α-subunit of the cardiac sodium channel, which plays a crucial role in determining the action potential of cardiomyocytes (40). Mutations in the *SCN5A* gene have been confirmed to be associated with DCM. These mutations can lead to abnormal sodium channel function, thereby affecting myocardial contraction (41). Pediatric patients with DCM carrying *SCN5A* mutations may present with symptoms such as ventricular enlargement, reduced contractile function, and arrhythmias (42, 43). Certain *SCN5A* mutations may also be associated with more severe disease progression and poorer prognosis, emphasizing the importance of early genetic testing (44).

*DSP* encode desmoplakin, a protein that plays a crucial role in cell-cell junctions. *DSP*-related DCM typically follows an autosomal dominant inheritance pattern, although autosomal recessive inheritance has also been reported (45, 46). Cardiomyopathy caused by *DSP* mutations is usually associated with arrhythmogenic right ventricular cardiomyopathy (ARVC) or DCM. DCM caused by *DSP* mutations may be accompanied by skin and hair abnormalities, such as curly hair and palmoplantar keratoderma (47). Although *DSP* mutation-related DCM can occur at any age, cases of childhood-onset have been increasingly reported (48).

*RBM20* is located on chromosome 10 and contains 14 exons. It encodes a protein of 1,227 amino acids that is primarily expressed in striated muscle, with the highest expression in cardiac muscle and almost no expression in non-muscle tissues. DCM-related *RBM20* gene mutations often occur in the arginine/serine-rich (RS) region, leading to loss of function in the expressed protein. Mutations in other regions occur less frequently than those in the RS region but can also reduce *RBM20* gene-splicing activity, affecting the expression of regulated genes and leading to DCM (49, 50). Pediatric patients with DCM carrying *RBM20* mutations may present with early onset heart failure and arrhythmias, particularly, an increased risk of ventricular arrhythmias (51–53).

Research has shown that most genes primarily cause DCM through autosomal-dominant inheritance patterns. Additionally, there have been a few reported cases of DCM caused by autosomal recessive, X-linked, or mitochondrial inheritance patterns (54).

Various methods have been applied to further explore new pathogenic genes and mutations. Currently, the main gene-testing strategies include: third-generation sequencing (NGS), genome-wide association studies (GWAS), whole-exome sequencing (WES), and whole-genome sequencing (WGS) (55, 56).

NGS is a targeted sequencing method that resolves genomic structural variations and complex regions. Customized DCM gene panels can cover 25–50 known DCM genes, including *MYH7, MYBPC3, TTN*, and *LMNA* (57). However, NGS cannot cover all known DCM genes and cannot detect new genes. GWAS can discover new gene loci associated with the disease by analyzing genotype and phenotype data from a large number of samples, but may miss the effects of rare variants (58). WES and WGS can rapidly identify rare pathogenic variants (59). Among these, WES is currently the preferred genetic diagnostic method for hereditary DCM. In addition, traditional methods, such as family linkage analysis, also play an important role in identifying pathogenic genes in familial DCM.

## Phenotypic diversity

Pediatric DCM exhibits significant genetic heterogeneity, and its clinical manifestations also show notable heterogeneity. The main phenotypic feature of DCM is myocardial injury, with the most prominent characteristics being significant dilation of the left ventricle or both ventricles and progressive decline in cardiac function (60). Some children experience symptoms of heart failure, including shortness of breath, fatigue, and reduced exercise tolerance (61). Additionally, some children may present with various types of common arrhythmias such as atrial fibrillation and ventricular tachycardia, which can potentially exacerbate symptoms or lead to sudden events (62).

In addition to cardiac manifestations, DCM can lead to multisystem involvement. When the nervous system is affected, it can cause motor disorders and cognitive decline (62). Studies have also found that some patients may exhibit skeletal muscle involvement such as muscle fatigue, weakness, and elevated muscle enzyme levels, providing an important perspective for understanding the role of the muscle-heart axis in hereditary diseases (63). Furthermore, DCM may indirectly affect the endocrine system, particularly through fluid and electrolyte imbalances caused by heart failure (64).

The phenotypic diversity of DCM is reflected in its pathogenic mechanisms, clinical manifestations, genetic background, and treatment responses, making it a complex and variable clinical challenge. Complex regulatory mechanisms underlie this process, such as the influence of genetic modifiers and environmental factors on the phenotype (65). A deeper understanding of the molecular mechanisms underlying the phenotypic heterogeneity of DCM will facilitate the development of targeted therapeutic approaches.

## Genotype-phenotype correlation studies

After reviewing a 76-year timeline in relation to studies on DCM-related gene mutations, the discovery of new DCM-related genes, gene-specific DCM outcomes, and insights into variant-environment interactions was found to have significantly advanced this field. The expansion of genomic phenotype analysis and integration of a series of prognostic factors into the variant environment are crucial (65). In Table 1, we summarized genotype and related phenotype in pediatric dilated cardiomyopathy.

### Different genotypes have different prognoses

Mutations in different genes can lead to variations in disease progression and prognosis. In pediatric DCM, *MYH7*-related DCM is characterized by early onset, high phenotypic expressivity, a low incidence of left ventricular reverse remodeling, and frequent progression to end-stage heart failure (66). Truncating mutations in *MYBPC3* result in loss of protein function,thereby accelerating the progression of cardiomyopathy, which may be significantly associated with disease severity and early onset (67). Patients with DCM carrying *FLNC* mutations may exhibit symptoms such as early onset heart failure and arrhythmia (68, 69). *TTN*tv mutations exhibit incomplete and age-dependent penetrance, with variable prognosis in affected children, reaching 100% penetrance by the age of 70 years; the same mutations have also been detected in unaffected relatives (70). Although *TTN*tv are less frequently found in pediatric cases, studies have shown a similar prevalence in adolescents and adults, suggesting that multiple clinical and genetic risk factors rather than a single *TTN*tv are required for its manifestation (71). Hasselberg et al. (33) found that *LMNA* mutation-related DCM is a highly pathogenic and age-dependent malignant disease, with affected children prone to arrhythmias and sudden death. These patients have a high incidence of adverse cardiac events and rapid disease progression, with some developing arrhythmias before left ventricular systolic dysfunction is observed. This indicates that genotype information can predict the clinical outcomes in affected children, aiding in the development of individualized treatment strategies.

### Heterogeneity exists in the same genotype

There may be significant differences in the phenotypic expression even among individuals carrying the same gene mutation. This phenotypic heterogeneity can be caused by various factors including environmental factors, epigenetic regulation, and genetic background (72). For example, Tharp et al. (17) found that the phenotype and severity of DCM are related to the location of *TTN*tv, with *TTN*tv mutations frequently occurring in the A-band region. In terms of sex, males carrying *TTN*tv were found to be associated with more severe clinical phenotypes and adverse clinical outcomes than females. Furthermore, patients with variants in the exon 11 region (c.2721–2760) of *RBM20* had a higher probability of developing DCM than those with variants in the exon 9 region (c.1881–1920) (51). Male carriers of *RBM20* variants were more likely to progress to end-stage heart failure than female carriers (73). However, sex differences were not significant in pediatric and adolescent cohorts. In addition, chemotherapeutic drugs can induce or exacerbate *TTN*tv mutation-related DCM in children. Nonsense variants of TTN are more common in patients with DCM, whereas frameshift termination and missense variants are more common in patients with neuromuscular and myocardial skeletal disorders (73).

### Genotype and electrocardiogram (ECG) abnormalities

Numerous children with DCM exhibit ECG abnormalities, such as T-wave changes, left bundle branch block, atrioventricular conduction abnormalities, and supraventricular arrhythmias (74). Children with ECG abnormalities have a higher mortality rate. Certain genetic causes of DCM result in malignant arrhythmic phenotypes. Autonomic nervous system imbalance and impaired myocardial repolarization homogeneity are two major underlying mechanisms of arrhythmia in patients with DCM (75). Laminopathies are often associated with prolonged PR intervals on ECG, which are indicators of cardiac conduction disorders. DCM associated with *DSP* mutations typically has a poor prognosis and may lead to malignant arrhythmias.

Patients with DCM carrying variants of *LMNA, PLN, FLNC*, and *RBM20* have an increased risk of developing arrhythmias. Younger patients with *LMNA*-related DCM and *RBM20*-related DCM show increased expression levels of arrhythmia-related cardiomyopathy. *LMNA*-related DCM exhibits a highly penetrant arrhythmic phenotype accompanied by multiple muscle involvement. Taylor et al. (76) reported that patients with *LMNA*-related DCM simultaneously experienced muscle involvement and arrhythmias, particularly chronic arrhythmias. *LMNA* variants account for up to 33% of DCM cases with atrioventricular conduction block (77). Similarly, patients carrying the *PLN* R14del founder variant typically develop ventricular arrhythmia and end-stage heart failure at a young age (78). Studies have found that left ventricular regional wall motion abnormalities are more common in *DSP*/*FLNC* genotypes, and these genotypes cause extensive regional left ventricular damage (79).

### Genotyping and neuromuscular diseases

The dilated phenotype is the most common cardiomyopathic manifestation in neuromuscular diseases (1). In pediatric neuromuscular diseases (NMD), particularly in dystrophies, abnormalities in the splicing of the CUG binding protein (CUG-BP) and muscle blind-like protein (MBNL) interfere with cellular signaling, resulting in toxic effects on muscle metabolism and RNA processing. This may cause changes in the cardiac structure and function, potentially resulting in the early onset of dilated cardiomyopathy (DCM) (80). Children with DCM who carry specific genotypes are prone to developing concurrent skeletal myopathies. Relevant neuromuscular symptoms in patients or relatives are important criteria for genetic analysis, because variants of *LMNA* are associated with DCM related to neuromuscular diseases (limb-girdle muscular dystrophy) (81). Emery-Dreifuss muscular dystrophy (EDMD) is closely related to dilated cardiomyopathy (DCM). In a study of 53 patients, mutations in *LMNA* were identified as a major cause, with 12 patients exhibiting significant cardiac involvement and 41 exhibiting muscle weakness. Therefore, screening for *LMNA* mutations is crucial in familial and sporadic cases associated with EDMD and DCM (82).

The impact of *TTN*tv extends beyond the heart, with 46%–57% of children with DCM and congenital heart disease exhibiting *TTN*-related neuromuscular diseases of varying severity (83). Sofie et al. (84) found that *TTN*tv carriers had a higher skeletal muscle fat content than patients with non-*TTN*tv hereditary DCM. Muscle biopsies in 62% of *TTN*tv carriers showed characteristics of skeletal muscle involvement, manifesting as well-aligned Z-lines and T-tubules, but with uneven and discontinuous M-lines, accompanied by excessive glycogen deposition. This was surrounded by autophagosomes, lysosomes, and mitochondrial autophagy with abnormal mitochondria, suggesting that patients with DCM with monoallelic *TTN*tv simultaneously have mild skeletal myopathy. Phenotype overlap may also occur in genes such as *DES* and *LMNA*, where DCM may be accompanied by skeletal myopathy (85).

## Treatment strategies and future research directions

Over the past few decades, while significant progress has been made in the treatment of DCM, many challenges remain. Transcription factors play a crucial role in dilated cardiomyopathy (DCM),may represent novel therapeutic targets.For instance, GATA4 and MEF2C are involved in the regulation of cardiac remodeling, and changes in their expression may lead to alterations in myocardial structure and function (86). Recent studies have also identified a correlation between mutations in the TBX5 gene and an increased risk of cardiac developmental defects and DCM. Additionally, TBX5 is associated with certain neuromuscular diseases, which often involve cardiac complications, including DCM (87, 88). Cardiovascular magnetic resonance can help identify the subclinical forms of the disease, facilitating a deeper understanding of the diverse phenotypes of DCM (89, 90) and the genes affecting pediatric DCM, with gene therapy offering a potential avenue for treating pediatric DCM (91). Cardiac function can be improved by modifying or replacing pathogenic genes in the cardiomyocytes to restore normal gene function. However, gene therapy is still in its early stages, and more clinical trials are necessary to verify the efficacy and safety of these therapeutic strategies (5).

Personalized medicine is becoming increasingly important in the treatment of pediatric DCM. Studies have indicated that patients with different phenotypes respond differently to specific treatments. For example, Verdonschot et al. (92) found differences in prognoses among different phenotype groups in DCM. Among the four phenotypic groups identified in their study, the arrhythmic group had the poorest prognosis and was highly susceptible to life-threatening malignant arrhythmias. After 12 months of regular drug therapy, 52.5% of patients experienced left ventricular reverse remodeling (LVRR), with the severe systolic dysfunction group showing the highest LVRR rate. Therefore, developing individualized treatment plans based on phenotypes and comorbidities of different patients can significantly improve treatment efficacy and patient quality of life.

Regarding future research directions, efforts should be made to expand sample sizes and establish more extensive genetic and phenotypic databases to achieve comprehensive assessments (93). At the same time, long-term follow-up of patients with DCM is crucial. Simultaneously,long-term follow-up allows for the evaluation of the effect of how different genotypes on disease progression and enables timely adjustments to treatment plans, thereby improving prognosis (94).

## Conclusion

Pediatric DCM exhibits significant genetic heterogeneity, with variations in the expression of multiple genes being closely associated with its development in childhood (2). These genes affect myocardial function through different pathways and can lead to diverse clinical phenotypes (65). This variability may result from the combined effects of gene-environment interactions, epigenetic regulation, and other unknown genetic factors (Figure 2). Personalized treatment plans targeting specific genotypes are promising for improving the prognosis and quality of life of affected children. The development of technologies such as GWAS and NGS has considerably advanced understanding of the action mechanisms of pediatric DCM (95). Gene therapy has shown promising results in animal models (96). However, their safety and efficacy require further validation through clinical trials. In the future, large-sample multicenter prospective cohort studies will help deepen the understanding of the genetic mechanisms underlying pediatric DCM.

In conclusion, research on the genotypes and phenotypes of pediatric DCM provides crucial perspectives and information for understanding its pathological mechanisms, thereby offering new hope for children with DCM. However, major challenges remain. We anticipate that as research progresses, these challenges can be addressed with the development of new therapeutic approaches for this complex disease.
